# Impact of Positive Lifestyle Behaviors on Direct Health Care Cost Savings for Low Back Pain

**DOI:** 10.1002/acr.25653

**Published:** 2025-12-14

**Authors:** Ye Tian, Katharine E. Roberts, Michelle Hall, Paula R. Beckenkamp, Angelo Sabag, Karoline Moe, Ana Paula Carvalho‐e‐Silva, Emily J. Callander, Paulo H. Ferreira

**Affiliations:** ^1^ School of Health Science, Faculty of Medicine and Health University of Sydney Sydney New South Wales Australia; ^2^ Sydney Musculoskeletal Health, The Kolling Institute University of Sydney Sydney New South Wales Australia; ^3^ Norwegian University of Science and Technology Trondheim Norway; ^4^ School of Public Health University of Technology Sydney Sydney New South Wales Australia

## Abstract

**Objective:**

This study aimed to investigate the relationship between a previously purpose‐developed lifestyle behavior scale and health care cost savings related to low back pain (LBP).

**Methods:**

This longitudinal study used data from the Australian Twin Back (AUTBACK) study. LBP and lifestyle behavior measures were collected at baseline. Physical activity (PA) was objectively quantified via an accelerometer. A lifestyle behavior scale was created using variables of body mass index, PA, smoking status, and sleep quality. Weekly health care use for LBP was collected over one year. Health care costs were calculated by aggregating expenses for health care visits and medications, encompassing personal and Australia Medicare costs, and analyzed by two‐part models.

**Results:**

Individuals with lower lifestyle behavior scores, women, and those with a baseline episode of LBP were more likely to incur health care use costs (n = 307). A total of 2.6% of participants accounted for over 56% of the total expenditures. A one‐point improvement in the lifestyle behavior scale was significantly associated with 23% decrease in overall health care costs for LBP (95% confidence interval [CI] 7%−36%; *P* = 0.006), 25% decrease in medication costs for LBP (95% CI 13%−35%; *P* < 0.001), and 27% decrease in health care visit costs for LBP (95% CI 14%−39%; *P* < 0.001). The predicted difference in yearly health care use costs between individuals with the lowest and highest lifestyle scores was AU$873.

**Conclusion:**

This study demonstrated the association between greater adherence to positive lifestyle behaviors and reduced health care costs related to LBP. Interventions aimed at improving lifestyle behaviors could yield substantial cost savings for individuals and the health care system, mitigating the burden of LBP.

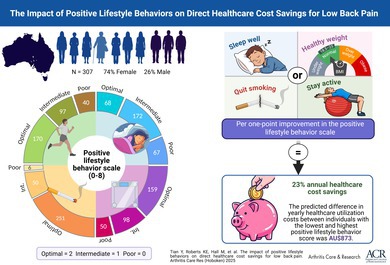

## INTRODUCTION

Low back pain (LBP) affects 619 million people globally and is the leading cause of years lived with disability (YLDs), accounting for a total of 69 million YLDs worldwide.^1^ Modifiable risk factors account for approximately two‐fifths of LBP‐related YLDs, with 12.5% of YLDs attributed to smoking, and 11.5% attributed to high body mass index (BMI).^1^ LBP also constitutes a significant economic burden on the individual and health care system. The average annual direct cost estimates, including costs for goods and health care services due to LBP, in high‐income countries range from US$4,671 to US$10,430 per patient with LBP.^2^ The Australian Institute of Health and Welfare reported that, in 2020 to 2021, Australians spent an estimated AU$3.4 billion on managing back problems,^3^ encompassing direct and indirect costs. Direct costs include expenses related to services and goods used in the diagnosis, management, and rehabilitation of LBP, whereas indirect costs represent the economic burden resulting from reduced work productivity and absenteeism.^2^

**SIGNIFICANCE & INNOVATIONS**

- The study demonstrates an association between greater adherence to positive lifestyle behaviors and reduced yearly direct health care costs due to low back pain (LBP). The potential savings can reach almost AU$900 in yearly overall health care use costs when individuals with lowest and greatest adherence to positive lifestyles are compared.
- A small subset (2.6%) of participants drove more than 56% of the total health care costs. This underscores the potential for targeted interventions focused on this subset of populations to yield substantial cost savings in LBP management.
- The study findings highlight the potential for self‐management and clinical and policy strategies promoting positive lifestyle behaviors to yield substantial cost savings in LBP management.

Lifestyle behaviors including smoking, alcohol consumption, and poor sleep have been found to be associated with higher health care use for LBP.^4^ A randomized controlled trial conducted in Australia found that interventions promoting healthy lifestyle behaviors, including weight loss and increased physical activity (PA), were associated with lower health care costs when compared with usual care (AU$708, 95% confidence interval [CI] AU$581–AU$850).^5^ A growing body of evidence supports the reallocation of health care resources from treatment of chronic conditions toward positive health initiatives or lifestyle interventions, with approaches such as health education and lifestyle changes resulting in improved health outcomes in chronic disease including cardiovascular disease, diabetes, and cancer.^6^ For instance, changes in lifestyles, including lowering energy intake via a healthy diet, and increasing energy expenditure via engagement in moderate intensity walking for 30 minutes five days per week, prevented the onset of type 2 diabetes by 58% for Americans at high risk.^7^ Interestingly, clusters of healthier lifestyles have also been found to be associated with lower levels of psychological distress, higher quality of life, and better self‐rated health.^8^

The novelty and use of a positive lifestyle behavior scale (PLBS) in LBP has been established in our previous study, with results revealing that increases in PLBS scores are associated with less health care usage for LBP over 12 months.^9^ Apart from the benefits in clinical outcomes, evidence linking healthy lifestyle factors to reduced health care costs for LBP could also encourage governments and policymakers to shift their focus toward positive health interventions, therefore leading toward long‐term cost savings and improved health outcomes at the individual and population levels. In the present study, we aimed to determine whether engagement in PA, sleep quality, smoking status, and BMI levels, measured by the PLBS, was associated with participants’ yearly direct health care use cost, including out‐of‐pocket expenses and items covered by Australia Medicare, due to LBP.

## MATERIALS AND METHODS

### Study design

Data for this study derived from the Australian Twin Back (AUTBACK) study, a longitudinal cohort study which aimed to evaluate the relationship between PA and LBP outcomes.^10^ The study was approved by the Twin Research Australia and the University of Sydney Human Ethics Committee (project 2015/407).

### Participants

Between 2015 and 2020, twins aged >18 years old (n = 401) were recruited across urban, remote, and rural Australia via Twins Research Australia. Eligible participants were twins aged >18 years old, with internet access via phone or computer and active email accounts. Individuals with self‐reported serious spinal pathology (eg, inflammatory, metastatic, or infectious disease of the spine), pregnant women, or those with a recent history of spinal surgery (≤12 months) were excluded. In the AUTBACK study, data were collected at baseline (ie, study enrollment) and six‐month follow‐up via self‐reported questionnaires (eg, demographic and anthropometric information, lifestyle behaviors, and LBP history) and accelerometer devices (eg, objective PA). Self‐reported measures including LBP symptoms (eg, pain severity, activity limitation), health care usen (eg, physiotherapist, surgery), self‐management strategies (eg, heat pack, light exercise) and medication (eg, over‐the‐counter medications, opioids) were collected weekly over 12 months.^10^ Details of the weekly electronic questionnaire are available in Supplementary A.1. In the current study, only participants who reported a lifetime prevalence of LBP (ie, LBP at any point in life) (Supplementary A.2) and provided both baseline data on BMI, smoking status, sleep variables, and PA levels and weekly data on health care utilization due to LBP were included. All participants provided informed consent before data collection.

### Assessment of exposures

A lifestyle behavior scale (PLBS) based on lifestyle factors including BMI, smoking status, PA, and sleep quality, which has been shown to predict health care usage related to LBP, was used to measure the level of healthiness in lifestyles.^9^ These four lifestyle factors were categorized as optimal (two points), intermediate (one point), or poor (zero points) based on prespecified thresholds to align with World Health Organization (WHO) recommendations and IPD‐Work (individual‐participant data meta‐analysis in working populations) Consortium articles.^11^, ^12^, ^13^ Equal weighting was used to avoid overemphasizing any single lifestyle factor. The individual scores were then summed into a composite scale ranging from zero (least adherent to positive lifestyle behaviors) to eight (most adherent to positive lifestyle behaviors). Details of the scoring system used for computing PLBS are available in Supplementary A.3.

BMI was calculated using weight and height measurements self‐reported at baseline. BMI between 18.5 (inclusive) and 25.0 kg/m^2^, indicating normal weight, was categorized as optimal; BMI between 25.0 (inclusive) and 30.0 kg/m^2^ was categorized as intermediate; BMI lower than 18.5 kg/m^2^ was categorized as underweight; and BMI of 30 kg/m^2^ and higher, indicating obesity, was categorized as poor.^11^ Baseline data on smoking status were collected, with nonsmokers categorized as optimal, exsmokers or occasional smokers categorized as intermediate, and current smokers categorized as poor. Participants wore an Actigraph accelerometer (GT1M/GT3X model) on their right hip for seven consecutive days during their waking hours. The methodology for processing PA variables can be found in Supplementary A.4. According to the WHO recommendations for PA,^12^ optimal levels were defined as either ≥150 minutes of moderate PA per week, ≥75 minutes of vigorous PA per week, or a combination of both. Moderate PA of 60 to 150 minutes or vigorous PA of 20 to 70 minutes per week were categorized as intermediate, and moderate PA of <60 minutes or vigorous PA of <20 minutes per week were categorized as poor.^13^, ^14^ The Pittsburgh Sleep Quality Index (PSQI), a standard clinical instrument with scores ranging from 0 to 21, was used to assess sleep quality.^15^ The sleep quality and daytime dysfunction subscales, each constituting one‐seventh of the PSQI, were used for the PLBS because they are thought to correlate with LBP prognosis. A sleep quality score of zero and daytime dysfunction score of zero or one were categorized as optimal, sleep quality score of one and daytime dysfunction score of one or two were categorized as intermediate, and sleep quality of two or three and daytime dysfunction of two or three were categorized as poor.^9^

### Covariates

The following variables were considered as possible covariates, given their potential to influence the prognosis of LBP and health care use due to LBP in previous studies: sex, age, depression, anxiety, stress, and a history of LBP in the previous four weeks.^16^, ^17^, ^18^ Depression, anxiety, and stress were assessed with 21‐item Depression Anxiety Stress Scales,^19^ Data on all covariates were collected via the baseline questionnaire.

### Assessment of health care use and health care costs due to LBP

Data on the number of LBP flare‐ups and care‐seeking behaviors associated with LBP were collected weekly, over 12 months, via electronic questionnaires (Supplementary A.1) sent via SMS or email. Each week with reported LBP was defined as a distinct LBP episode, for which the corresponding health care use cost was ascertained. Overall health care use cost due to LBP was calculated by aggregating the total expenses incurred from health care visits and medication usage for LBP over a 12‐month period. The health care costs encompass both out‐of‐pocket expenses and items covered by Medicare, the government‐funded health care insurance program in Australia.

A hierarchical decision process was employed when calculating health care visit and medication costs. Health care services were valued using unit service prices of the Australian Medicare Benefit Schedule (MBS).^20^ Medication usage were valued by (1) applying the conservative daily dosages based on standard Australian medication guideline^21^ to the reported days, (2) annualizing quantities and rounding to whole package sizes, and (3) applying unit prices of the Australian Pharmaceutical Benefits Scheme (PBS).^21^ If the MBS or PBS unit prices were unavailable, prices were obtained by computing the average prices from the top five to 10 relevant private clinic services or pharmacy items in two major Australian states, Victoria and New South Wales. All health care costs are reported in Australian dollars (AU$1 = US$0.656 or €0.606) and reflected the costs of goods and services in the 2023 to 2024 financial year.

### Statistical analysis

Descriptive statistics were conducted and presented as medians and interquartile ranges (IQR) due to non‐normal distribution of the data. For variables presenting frequently zero values, mean and SD was reported. Based on previous research, due to the large fraction of observations with zero expenditures, a two‐part model was used to calculate adjusted associations between the PLBS and the primary outcome. In the two‐part model, logistic regression (logit) was used to model the probability that a person has any health care expenditures using the full sample.^22^ Then, we modeled the association between PLBS and the primary outcome with a generalized linear model (GLM), interoperating a γ‐distribution and a log‐link function, on the subset of people who had any expenditures.^22^ Specifically, to quantify the economic impact of lifestyle behaviors, we calculated the adjusted mean annual LBP‐related health care use costs across PLBS levels (0–8) using the “margins” command following the two‐part model. The margins command computed unconditional marginal predictions by evaluating costs at each integer PLBS value while preserving observed covariate distributions, thereby accounting for cohort heterogeneity. Adjusted models accounted for the potential confounding effects of sex, age, depression, anxiety, stress, and a history of LBP in the previous four weeks at baseline. Adjusted odds ratios (ORs), cost ratios (CRs), and 95% CIs were used to quantify the associations between the exposure variables and the study outcome. To account for the nonindependence of data from complete twin pairs, robust standard errors were calculated using the “vce cluster” command, which clusters on the identifier distinguishing twins within a pair. Statistical significance was set at *P* < 0.05. All analyses were performed using Stata (version 18).^23^

### Data sharing

The dataset generated during and/or analyzed during the current study are included in the article or uploaded as online supplementary information. Deidentified individual participant data will be made available, including data dictionaries, for approved data sharing requests. Individual participant data will be shared that underlie the results reported in this article, after deidentification and normalization of information (text, tables, figures, and appendixes). Study protocol has been published and is publicly available. Anonymized data will be available beginning three months after and ending 10 years after publication of this article to researchers who provide a completed Data Sharing Agreement that describes a methodologically sound proposal for the purpose of the approved proposal.

## RESULTS

**Figure 1:**
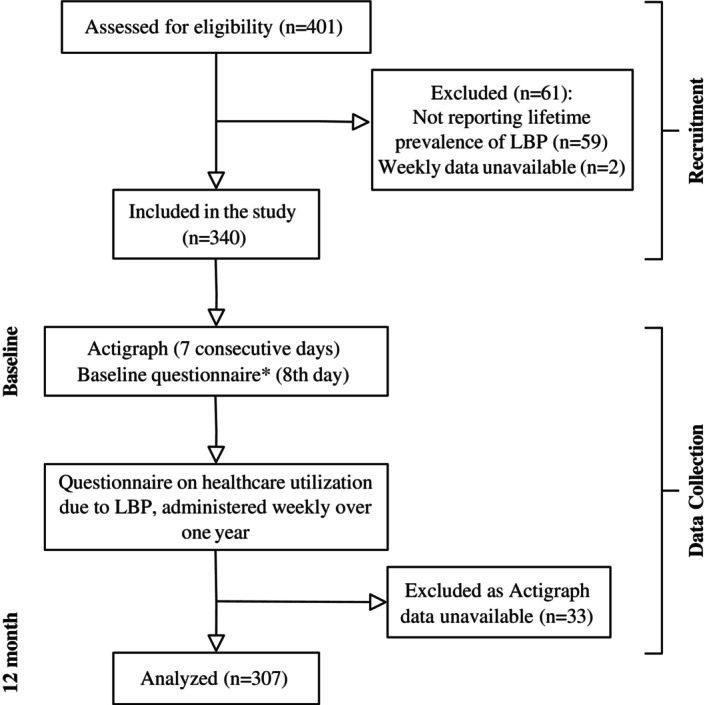
Study flowchart. *Exposure variables were collected via the baseline questionnaire, including body mass index, smoking status, sleep quality, and physical activity. Covariates were collected via the baseline questionnaire, including sex, age, depression, anxiety, stress, and a history of LBP in the previous four weeks. LBP, low back pain.

Overall, 16,690 weekly questionnaires assessing LBP status and health care use costs due to LBP were completed across 401 eligible participants, corresponding to a response rate of 80% (16,690 questionnaires received out of 20,852 [401 participants × 52 weeks]). A total of 59 participants who did not report a lifetime prevalence of LBP were excluded. Only two participants were excluded due to an unavailable weekly dataset, resulting in minimal impact on study generalizability. A total of 33 participants were excluded from the analyses due to unavailable Actigraph data (Figure 1). No multiple imputation was conducted in the analyses.

### Baseline characteristics

**Figure 2:**
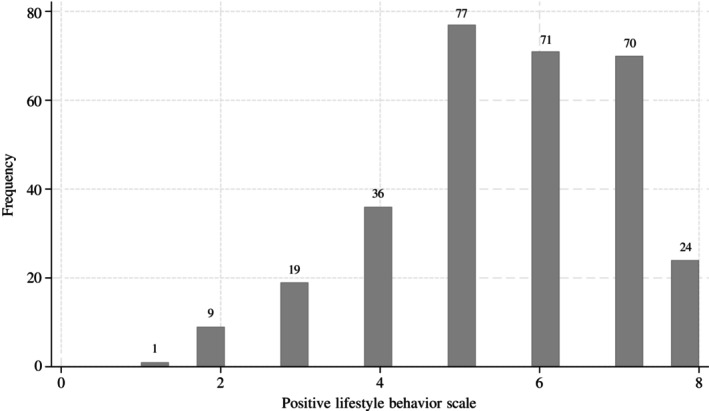
Histogram showing the distribution of positive lifestyle behavior scale.

A total of 307 participants were included in the current study. Over the 12‐month study period, participants collectively reported 3,094 weeks or 11,334 days with LBP. On average, each participant experienced LBP for 10.1 days (SD 12.7). The baseline characteristics of the sample are presented in Table 1. The median age was 56 years (IQR 45.0–62.3), and most participants (74%) were women. A total of 48% of the study sample reported a recent episode of LBP in the four weeks before baseline completion; 34% (47 of 139) of those participants reported experiencing LBP for six weeks or less, 4% (6 of 139) reported experiencing LBP for six weeks to three months, and 62% (86 of 139) reported experiencing LBP for three months or more. The median BMI was 24.6 kg/m^2^ (IQR 22.1–28.1), and 52% of participants had BMI between 18.5 and 24.9 kg/m^2^ and were categorized as having optimal BMI. A total of 55% of participants were categorized as having optimal PA (ie, moderate PA of ≥150 minutes and/or vigorous PA of ≥75 minutes per week). Although only 22% of participants were categorized as having optimal sleep status (ie, great sleep quality and minimal daytime dysfunction), 82% of participants were nonsmokers and categorized as having optimal smoking status. The median PLBS was six (IQR 5–7), with the majority of participants (71%) scoring five, six, or seven (Figure 2). A total of 8% of the participants (n = 24) scored the maximum PLBS of eight, no participant presented the minimum score of zero, and only one participant (0.3%) scored one.

**Table 1:** Baseline characteristics of the participants*

Characteristic	n	Values
Age, median (IQR)	307	55.8 (45.0–62.3)
Female, %	307	74^a^
Body mass index, median (IQR), kg/m^2^	307	24.6 (22.1–28.1)
Zygosity (monozygotic), %	307	66^b^
Recent episode of LBP (yes)^c^, %	307	48^d^
LBP duration^e^, %		
≤6 weeks	47	34
6 weeks to 3 months	6	4
≥3 months	86	62
Depression (0–42)^f^, median (IQR)	307	2 (0–4)
Anxiety (0–42)^f^, median (IQR)	307	2 (0–4)
Stress (0–42)^f^, median (IQR)	307	6 (2–12)
Positive lifestyle variables, %		
BMI categories		
Optimal	159	52
Intermediate	98	32
Poor	50	16
Smoking status categories, %		
Optimal	251	82
Intermediate	50	16
Poor	6	2
Sleep categories, %		
Optimal	68	22
Intermediate	172	56
Poor	67	22
Physical activity categories^g^, %		
Optimal	170	55
Intermediate	97	32
Poor	40	13
Positive lifestyle behavior scale, median (IQR)	307	6 (5–7)
1, %	1	0.33
2, %	9	2.93
3, %	19	6.19
4, %	36	11.73
5, %	77	25.08
6, %	71	23.13
7, %	70	22.80
8, %	24	7.82

* BMI, body mass index; IQR, interquartile range; LBP, low back pain.

^a^ n = 226.

^b^ n = 204.

^c^ Recent episode of LBP is defined as experiencing low back pain ≤4 weeks before completion of baseline assessment.

^d^ n = 146.

^e^ Data on LBP duration were collected when individuals reported recent episode of LBP.

^f^ 21‐Item Depression Anxiety Stress Scale; each of the three domains range from 0–42, with higher scores representing higher levels of each domain.

^g^ Device‐based measures; values are minutes/week, assessed with an accelerometer.

### The impact of PLBS on health care costs for LBP

The median overall health care cost due to LBP over 12 months was AU$15.78 (IQR 0–110.35), with the highest median cost of AU$41.39 (IQR 15.78–528.18) when PLBS was scored at three. Those who scored one, seven, or eight had an overall health care cost of AU$0 (Table 2). Out of 307 participants, 173 (56%) had an overall health care cost over AU$0. The primary health care visit cost driver was physiotherapy visits (mean AU$67.48, SD AU$462.03), and the primary medication cost drivers were usage of weak opioids (mean AU$18.19, SD AU$117.75) and nonopioids (mean AU$11.10, SD AU$22.17). Only 2.6% of participants (n = 8) had an overall health care cost higher than AU$1,000, and 1.3% of the participants (n = 4) had an overall health care cost exceeding AU$4,000. Details of the distribution and breakdown of health care use costs are available in Supplementary Figure 1 and Supplementary Tables 1 and 2.

**Table 2:** Descriptive analysis of positive lifestyle behavior scale and overall health care use costs due to low back pain*

Positive lifestyle behavior scale^a^	n	Cost^b^_,_ median (IQR)	Cost, mean (SD)
Total	307	15.78 (0–110.35)	212.36 (891.37)
0	0	N/A	N/A
1	1	0 (0–0)	0 (N/A)
2	9	15.78 (0–15.78)	15.78 (24.95)
3	19	41.39 (15.78–528.18)	1,100.41 (2,483.28)
4	36	15.78 (0–119.24)	380.36 (1,606.31)
5	77	15.78 (0–102.51)	152.82 (442.49)
6	71	15.78 (0–252.25)	161.45 (279.60)
7	70	0 (0–82.90)	86.88 (186.25)
8	24	0 (0–15.78)	47.49 (142.25)

* IQR, interquartile range; N/A, not applicable.

^a^ Total lifestyle behavior scale ranges from 0 to 8 (where 0 represents the lowest positive lifestyle behavior scale, and 8 represents the highest positive lifestyle behavior scale).

^b^ Overall health care use cost is defined as the total expenses incurred from health care practitioner visits and medication usage over the 12‐month study period.

Residual plot (Supplementary Figure 2) indicated linearity across 96.7% of the PLBS distribution (scores ≥2), with minor deviations only at the lower extreme (scores ≤2; n = 10, 3.3% of observations). Given both the limited representation of PLBS of two and lower and the absence of meaningful deviation from linearity above this threshold, we kept the PLBS variable as a continuous linear variable in all analyses.

In the full sample (n = 307), the association between PLBS and the probability of having health care expenditures larger than AU$0 is presented in Table 3 (logit column). Higher scores on the PLBS were significantly associated with a lower probability of incurring overall health care costs (OR 78%, 95% CI 65%–94%; *P* = 0.008) and a decreased probability of incurring medication costs (OR 79%, 95% CI 66%–94%; *P* = 0.008). This indicates that an increase in PLBS was associated with a decreased chance of incurring any expenditures (>AU$0) on LBP.

**Table 3:** The association between the positive lifestyle behavior scale, covariates and overall costs, medication costs, and health care visit costs due to low back pain*

Exposure	Logit (n = 307)		GLM (n = 173)	
	OR (95% CI)	*P* value	CR (95% CI)	*P* value
Positive lifestyle behavior scale^a^				
Overall health care costs^b^	0.783 (0.654–0.938)	0.008	0.771 (0.641–0.927)	0.006
Medication costs^b^	0.789 (0.662–0.940)	0.008	0.754 (0.650–0.874)	<0.001
Health care visit costs^b^	0.943 (0.788–1.129)	0.52	0.727 (0.614–0.860)	<0.001

* CI, confidence interval; CR, cost ratio; GLM, generalized linear model; OR, odds ratio.

^a^ Positive lifestyle behavior scale was treated as a continuous variable in these analyses.

^b^ All analyses were adjusted for sex, age, depression, anxiety, stress, and recent episode of low back pain at baseline.

In the subset of people who had any expenditures due to LBP (n = 173), the association between PLBS and overall health care costs, medication costs, and health care visit costs due to LBP is presented in Table 3 (GLM column). Among those who spent more than AU$0 due to LBP, a one‐point improvement in the PLBS was significantly associated with a 23% decrease in overall health care costs (CR 0.77, 95% CI 0.64–0.93; *P* = 0.006), a 25% decrease in medication costs (CR 0.75, 95% CI 0.65–0.87; *P* < 0.001), and a 27% decrease in health care visit costs due to LBP (CR 0.73%, 95% CI 0.61–0.86; *P* < 0.001). Being a man was significantly associated with a 76% lower overall health care costs (CR 0.24, 95% CI 0.11–0.54; *P* < 0.001) compared with being a women (Supplementary Table 3). Having a baseline episode of LBP was significantly associated with a 208% increase in overall health care costs (CR 3.08, 95% CI 1.87–5.07; *P* < 0.001) (Supplementary Table 3).

### Predicted health care use costs

**Figure 3:**
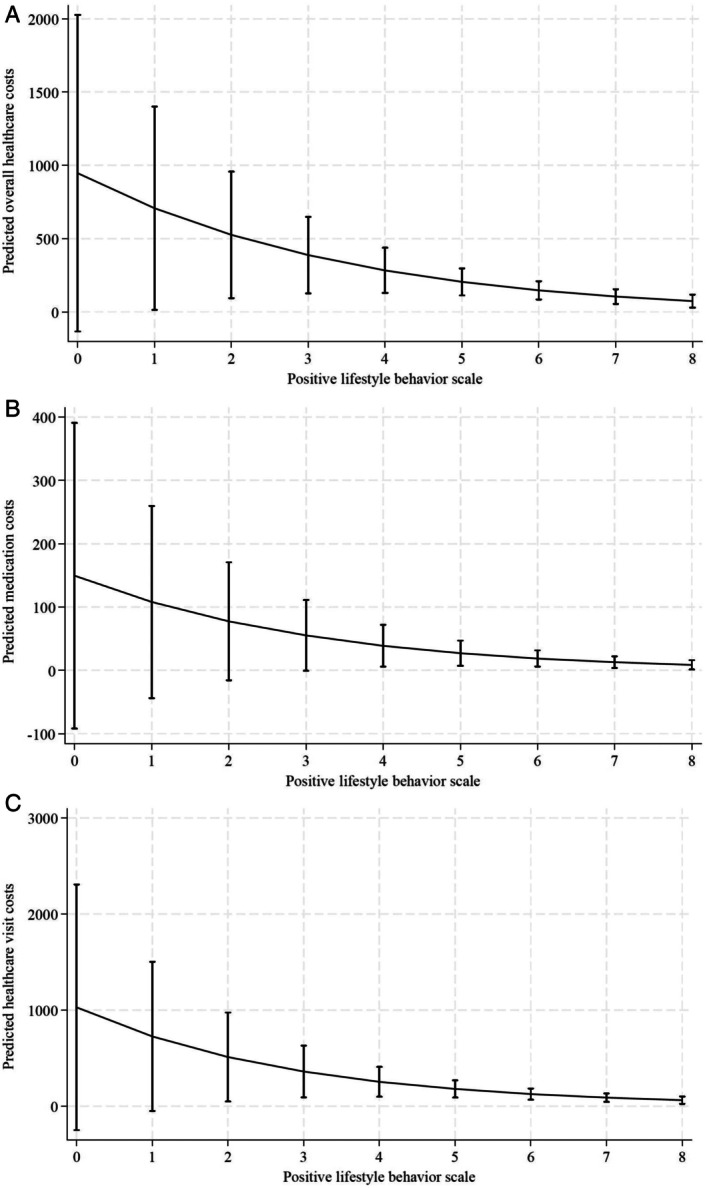
Margins plot showing the predicted health care use costs with confidence interval at each level of positive lifestyle behavior scale for: (A) overall health care costs, (B) medication costs, and (C) health care visit costs due to low back pain.

For participants with the median (six) PLBS, the predicted overall health care costs over 12 months was AU$147.30 (95% CI 85.00–209.60; *P* < 0.001). The predicted overall health care costs were highest (AU$946.49; 95% CI −133.66 to 2026.65; *P* = 0.086) for those with the minimum (zero) PLBS. On the other hand, those who scored eight on the PLBS had the lowest predicted overall health care costs of AU$73.34 (95% CI 28.81–117.87; *P* = 0.001) (Figure 3; Supplementary Table 4). The predicted difference in overall health care costs between individuals with the lowest and highest lifestyle scores was AU$873 (Supplementary Table 4). For participants with the median (six) PLBS, the predicted medication costs were AU$18.70 (95% CI 5.98–31.42; *P* = 0.004), and the predicted health care visit costs were AU$125.39 (95% CI 67.12–183.67; *P* < 0.001). For participants with the minimum (zero) PLBS, the predicted medication costs (AU$149.42; 95% CI −91.83 to 390.67; *P* = 0.225) and predicted health care visit costs (AU$1,028.44; 95% CI −250.09 to 2,306.98; *P* = 0.115) were highest (Figure 3; Supplementary Table 4).

## DISCUSSION

In the present study, we observed positive associations between people's positive lifestyle behaviors, assessed by the PLBS, and a reduction in their annual health care use costs—such that optimal lifestyle behaviors were associated with significantly lower overall health care, medication, and health care visit costs for LBP. These costs were primarily derived from savings associated with physiotherapy sessions, as well as medication usage, such as weak opioids and nonopioids. As individuals adopt more positive lifestyle behaviors, the predicted annual health care costs associated with LBP demonstrated a corresponding decrease. These findings suggest that for individuals with a lifetime history of LBP, adherence to positive lifestyle behaviors may lead to significant cost savings in annual health care expenditures related to LBP. For governments and policymakers, these findings underscore the importance of increasing access to positive lifestyle interventions and promoting positive health initiatives.

In line with existing evidence,^18^, ^24^ 56% of the study participants, with a lifetime history of LBP, chose to seek care and pay for LBP treatment. Despite the burden of LBP, approximately 56% to 60% of people suffering from LBP seek health care.^18^, ^24^ This low health care–seeking rate is potentially due to the self‐limiting nature of acute episodes of LBP, which often resolves with self‐care strategies.^24^ In the current study, yearly health care cost savings incurred from health care professional visits and medication usage were strongly associated with positive lifestyle behaviors, including healthy BMI, minimal smoking, increased PA, and optimal sleep quality. A one‐point improvement in people's lifestyle behavior score was significantly associated with a 23% decrease in their overall health care costs, a 25% decrease in medication costs, and a 27% decrease in health care visit costs due to LBP. These findings are in line with a previous interventional study showing that weight loss and optimal PA adherence resulted in AU$708 (95% CI 581–850) lower annual health care costs in adults with chronic LBP when compared with usual care.^5^ In our study, men, compared with women, used 76% lower overall health care costs. This finding is consistent with a previous meta‐analysis, in which pooled results showed that sex is an important determinant in the care‐seeking behavior.^18^ Sex differences in costs may reflect both biologic factors (eg, higher LBP prevalence in women) and sociocultural influences (eg, caregiver roles traditionally being adopted by women). According to the 2023 Global Burden of Disease Report, the global LBP prevalence rates were higher among women compared with men across all age groups.^1^ Compared with men, women have worse self‐perceived health, which could account for their greater use of health care services, such as primary care visits and use of diagnostic procedures.^25^ Moreover, women frequently assume the caregiver roles of the family and, therefore, are more heightened in health awareness and active in care‐seeking behaviors.^26^ These sex differences underscore the need to adopt sex sensitivity into health care practice by raising awareness in public health, enhancing clinical understanding, and developing skills among health professionals^27^ to ensure equitable care delivery while addressing potentially modifiable cost drivers.

Only a small proportion of the sample (2.6%) had an overall health care cost higher than AU$1,000, and 1.3% had an overall health care cost higher than AU$4,000. Individuals who spent over AU$1,000 annually on LBP care accounted for >56% of the total expenditure within the study sample. These findings suggest that a relatively small subset of the population drives most health care expenditure related to LBP. Participants that drove the higher costs (ie, that spent over AU$4,000) were women, had relatively low PLBS scores (median 3, IQR:3–3.5), most commonly saw the physiotherapist of all health care practitioners, most commonly used weak opioids of all medications for LBP, and all had three months or more of LBP duration and higher stress level (Supplementary Table 2). A previous study and a systematic review showed that, rather than socioeconomic status, factors that increased care‐seeking behaviors for LBP were older age, female sex, higher grades of pain and disability, and fear of the impact of pain on participation with usual social roles.^18^, ^28^ Policymakers could target those with higher risks for health care costs by promoting specific health care plans, including community‐based exercise and disability benefit and support programs.

Although people who adopt healthier lifestyle behaviors might not spend less time suffering from activity limiting LBP,^9^ they are likely to use less health care and increase their cost savings related to LBP. Simple lifestyle changes, such as walking in the community, healthier eating, quitting smoking, or improving sleeping habits, could help individuals to significantly reduce the financial burden of LBP management. For individuals with LBP and clinicians who manage LBP, incorporating lifestyle programs could empower patients to adopt diverse self‐care strategies, improve LBP health outcomes, and increase cost savings. Components of lifestyle programs identified for addressing LBP include healthy diet, exercise and leisure‐time PA, sleep hygiene, stress resilience (eg, mindfulness, diaphragmatic breathing), reduced substance use (eg, nonsmoking status, moderate alcohol consumption), social connection (eg, community activities, exercise buddy), and self‐management strategies (eg, self‐massage, flare‐up management).^29^ The effectiveness of these interventions can vary depending on factors like individual preferences, cultural contexts, and socioeconomic conditions. Therefore, a patient‐centered approach is necessary to tailor lifestyle interventions to specific populations and maximize the positive impact over time.

For policymakers, prioritizing healthy lifestyle interventions via advocating positive lifestyle behaviors plays a crucial role in promoting overall health, enhancing quality of life by preventing chronic disease, reducing disability level, and alleviating health care economic burdens at both an individual and societal level. For instance, the Australian National Tobacco Strategy 2023 to 2030 is in place to reduce the use of both tobacco and e‐cigarettes.^30^ Many Australians consume excessive energy through unhealthy diets.^31^ The WHO advises that reasonable price policies on sugar‐sweetened beverages and subsidies for fresh fruits or vegetables are effective in improving the affordability and consumption of healthier food products, hence improving BMI‐related health outcomes.^32^ In Newfoundland and Labrador, Canada, the Physical Activity Tax Credit offers a refundable tax credit of up to CA$2,000 per household, which acts as an incentive for families as they access sport and leisure‐time PA.^33^ Although a previous study has shown that national health campaigns have a beneficial impact on the adoption of various positive lifestyle behaviors,^34^ policymakers should also acknowledge, and target, potential barriers in the implementation of healthy lifestyle interventions, for example, by improving accessibility and affordability to positive lifestyle services (eg, Healthdirect, Alcohol and Drug Information Hotline, community‐based exercise programs) and raising public awareness via empowering patient education. People are more likely to make healthy lifestyle choices when they are enabled and empowered with the necessary knowledge, motivation, and support.

The AUTBACK study included participants across all regions of Australia, and the response rate was high (80%), providing a large nationwide database. The longitudinal nature of the study allowed us to collect repeated weekly measures of health care use over a 12‐month period. Additionally, device‐based measures of PA were used, which demonstrated acceptable reliability and validity for assessing PA levels.^35^ Whereas earlier work established that healthier lifestyles reduced health care usage frequency,^9^ our study moved beyond evenly weighted health care usage counts (eg, one visit to physiotherapist or one opioid consumption) and uniquely quantified the substantial cost saving, accounting for varied service and good prices, and identified high‐cost subgroups for optimized resource allocation. For example, our analysis revealed that reduced health care use translated to a maximum predicted cost saving of AU$873.15 per patient annually when accounting for service‐specific cost variations. Furthermore, our identification of the distinct cost‐profile subgroups (ie annual health care utilization cost larger than AU$4,000) reported in Supplementary Table 2 enables health care systems to target populations in which lifestyle modifications yield the greatest economic returns.

Less than 10% of the eligible participants (n = 33 of 340; 9.7%) were excluded from the analyses because their Actigraph data was not available. Additionally, data on alcohol consumption and diet were not available in our study. The number of people who scored zero to three on the PLBS was low in our study sample, which may affect the generalizability of the study findings to people with worse lifestyle behaviors. Exploration of the characteristics in individuals with higher annual health care expenditures was also limited by the small subgroup size. Although we adjusted the analyses for participants’ report of a recent history of LBP at baseline, and long‐term health care cost patterns were captured repetitively for 12 months, reverse causality remains plausible: LBP symptoms may in fact impact lifestyle behaviors (eg, reducing PA or sleep quality), therefore inflating their association with health care costs. Future studies should consider the use of repeated measures of more comprehensive lifestyle factors and include larger samples to further investigate the characteristics and health care use patterns of individuals with lower PLBS and higher health care expenditures associated with LBP. Also, the inclusion of a sample of participants with no history of LBP at baseline who are followed longitudinally until an episode of LBP is observed would be advised, although finding individuals who have never experienced LBP is extremely difficult given the prevalence of the condition.

To conclude, this study demonstrated clear associations between the adoption of positive lifestyle behaviors and a reduction in health care use costs related to LBP. Self‐managing and clinical and political interventions aimed at improving the adoption and adherence to positive lifestyle behaviors could potentially yield substantial cost savings for individuals and the health care system while mitigating the burden of LBP.

## AUTHOR CONTRIBUTIONS

All authors contributed to at least one of the following manuscript preparation roles: conceptualization AND/OR methodology, software, investigation, formal analysis, data curation, visualization, and validation AND drafting or reviewing/editing the final draft. As corresponding author, Ms Tian confirms that all authors have provided the final approval of the version to be published, and takes responsibility for the affirmations regarding article submission (eg, not under consideration by another journal), the integrity of the data presented, and the statements regarding compliance with institutional review board/Declaration of Helsinki requirements.

## Supporting information


**Disclosure form**.

**Appendix S1:** Supplementary Appendix
