# Supplementary material for: Impact of Positive Lifestyle Behaviors on Direct Health Care Cost Savings for Low Back Pain
Source: Arthritis Care Res (Hoboken). 2025 Dec 14;78(4):537–46. doi: 10.1002/acr.25653 (PMC13034093; doi:10.1002/acr.25653)
Supplement: Supplementary file 2 — Appendix S1: Supplementary Appendix [file ACR-78-537-s001.docx]

**Supplementary appendix**

| **Items** |  |  |  |  | |
| --- | --- | --- | --- | --- | --- |
| **Supplementary A.** Details of study design | | | | |  |
| **Supplementary A.1** Assessment of healthcare utilization and healthcare costs due to LBP | | | | |  |
| **Supplementary A.2** Assessment of lifetime prevalence of LBP | | | | |  |
| **Supplementary A.3** Scoring system used for computing positive lifestyle behavior scale | | | | |  |
| **Supplementary A.4** Assessment and methodology for processing physical activity variables | | | | |  |
| **Supplementary B.** Distribution of data for the primary outcome | | | | |  |
| **Supplementary Figure 1.** Histogram showing the distribution of overall healthcare utilization cost  **Supplementary Figure 2.** Lowess-smoothed residual plot assessing linearity of positive lifestyle behavior scale in the two-part cost model | | | | |  |
| **Supplementary Table 1.** Descriptions of the overall healthcare utilization cost  **Supplementary Table 2.** Characteristic of the participants having overall healthcare utilization cost larger than AU$4,000 | | | | |  |
| **Supplementary C.** Results of the two-part model and margins prediction | | | | |  |
| **Supplementary Table 3.** The association between the positive lifestyle behavior scale, covariates and overall costs, medication costs and healthcare visit costs due to low back pain | | | | |  |
| **Supplementary Table 4.** Margins predictions for overall costs, medication costs and healthcare visit costs | | | | |  |

**Supplementary A. Details of study design**

## A.1 Assessment of healthcare utilization and healthcare costs due to LBP

Data on the number of LBP flare-ups and care-seeking behaviors associated with LBP were collected weekly, over 12 months, via electronic questionnaires (weekly low back pain questionnaire) which were sent to participants via SMS or email. Firstly, participants were asked whether they had experienced LBP in the past week (yes/no). Those who responded ‘yes’ were asked whether they sought care from healthcare practitioners, used any medication for their LBP (yes/no). Those who answered ‘yes’ were asked to specify the types of healthcare practitioner (e.g., general practitioner, physiotherapist, chiropractor, surgical procedure, emergency department, or other), the types of LBP medications (e.g., non-opioid medication, weak opioid medication, strong opioid medication, antidepressant medication, or other medication); and the frequency (counts) of days for each type of care-seeking behavior over the past week.

**Weekly low back pain questionnaire**

1. Have you had low back pain in the last 7 days?

( ) Yes

( ) No

*Observation: the following questions will only appear if the participant has answered “Yes” for the previous question.*

Please, only answer questions 2, 3 and 4 if your low back pain started in the past week.

1. Could you please describe the day and time you first noticed your low back pain this week:
2. Could you please recall whether you were doing any of these tasks two hours before your low back pain started?

( ) Lifting, lowering, or pushing an object

( ) Bending down

( ) Lifting a child or an animal

( ) A vigorous activity such as running, using heavy tools

( ) A moderate activity such as cycling, general home repairs

( ) A trip, slip or a fall

( ) Other: Please specify:

( ) None.

1. Could you please recall whether you were doing any of these tasks last week (7 days ago), when you did not have back pain, at the same time of your low back pain onset this week?

( ) Lifting, lowering, or pushing an object

( ) Bending down

( ) Lifting a child or an animal

( ) A vigorous activity such as running, using heavy tools

( ) A moderate activity such as cycling, general home repairs

( ) A trip, slip or a fall

( ) Other: Please specify:

( ) None.

1. For how many days have you had low back pain in the last 7 days?

( ) 1

( ) 2

( ) 3

( ) 4

( ) 5

( ) 6

( ) 7

1. Please indicate on average what was the intensity of your low back pain on a scale from 0 to 10, where 0 means “no pain” and 10 means “the worst pain imaginable”, over the last 7 days.

| 0 | 1 | 2 | 3 | 4 | 5 | 6 | 7 | 8 | 9 | 10 |
| --- | --- | --- | --- | --- | --- | --- | --- | --- | --- | --- |
| No pain |  |  |  |  | Moderate Pain |  |  |  |  | Worst possible pain |

1. Was the low back pain bad enough to limit any of the following activities **last 7 days** (select as many as applicable)?

( ) Work

( ) Socializing

( ) Sports

( ) Hobbies

( ) Intimacy

( ) Chores

( ) I did not have any activities limited because of my pain

For how many days was the low back pain bad enough to limit your activities? (this question will appear for each option selected by the participant)

( ) 1

( ) 2

( ) 3

( ) 4

( ) 5

( ) 6

( ) 7

1. Have you sought any of the following treatments for this low back pain **last 7 days** (select as many as applicable)?

( ) GP

( ) Physiotherapist

( ) Chiropractor

( ) Emergency department

( ) Surgical procedure

( ) Other Please specify:

I have treated myself with (does not include medication use / select as many as applicable):

( ) heat pack

( ) bed rest

( ) light exercise, such as walking

( ) hot shower

( ) by seeking information on internet and books

( ) None of the mentioned above

Trials used written infor-

mation, discussion sessions, and audiovisual resources

(i.e., audiotape, videotape, or web site) as self-management

strategies.

( ) I did not seek any treatment for my pain

For how many days did you have to seek treatment for your low back pain? (this question will appear for each option selected by the participant)

( ) 1

( ) 2

( ) 3

( ) 4

( ) 5

( ) 6

( ) 7

1. Have you taken any of the following medications for you low back pain **last 7 days** (select as many as applicable)?

( ) Non-Opioid Analgesics (Examples: Paracetamol, Aspirin, Nurofen, Voltaren)
( ) Weak Opioid Analgesics (Examples: Codeine, Oxycodone, Tramadol)

( ) Strong Opioid (Example: Morphine)

( ) Antidepressants (Examples: Zoloft, Prozac, Efexor, Allegron, Avanza, Edronax)

( ) Natural pain relievers

( ) Others Please specify:

( ) I did not take any medication for my pain.

For how many days did you have to take medication for your low back pain? (this question will appear for each option selected by the participant)

( ) 1

( ) 2

( ) 3

( ) 4

( ) 5

( ) 6

( ) 7

## A.2 Assessment of lifetime prevalence of LBP

At baseline, lifetime prevalence of LBP was assessed with the question: “In your lifetime, have you ever had pain in your low back?” (yes/no).

**A.3 Scoring system used for computing positive lifestyle behavior scale**

| **Positive lifestyle behavior scale** | | |
| --- | --- | --- |
| **BMI** | | |
| 2 | Optimal | ≥18.5 and <25 (normal weight) |
| 1 | Intermediate | ≥25 and <30 (overweight) |
| 0 | Poor | ≥30 or <18.5 (underweight or obese) |
| **Smoking** | | |
| 2 | Optimal | Nonsmoker |
| 1 | Intermediate | Former or occasional smoker |
| 0 | Poor | Current smoker |
| **Physical Activity** - Actigraph accelerometer (GT1M/GT3X model) | | |
| 2 | Optimal | ≥150 minutes of moderate or ≥75 minutes of vigorous activity |
| 1 | Intermediate | 60−150 minutes of moderate or 20 −70 minutes of vigorous activity |
| 0 | Poor | <60 minutes of moderate or <20 minutes of vigorous activity |
| **Sleep** - PSQI | | |
| 2 | Optimal | Optimal sleep quality = 0 and daytime dys-function = 0 or 1 |
| 1 | Intermediate | sleep quality = 1 and daytime dysfunction = 1or 2 |
| 0 | Poor | sleep quality = 2 or 3 and daytime dysfunction = 2 or 3 |
| Total lifestyle behavior scale ranges from 0 to 8 (where 0 represents the lowest positive lifestyle behavior scale and 8 represents the highest positive lifestyle behavior scale). BMI: body mass index; PSQI: Pittsburgh Sleep Quality Index. | | |

**A.4 Assessment and methodology for processing physical activity variables**

Participants were asked to wear an Actigraph accelerometer (GT1M/GT3X model), secured to their waist above the right hip via an elastic belt, for seven consecutive days during their waking hours, except while swimming or showering. Accelerometer data was processed using the manufacturer’s software (ActiLife). Freedson Combination (1998) and Freedson Adult (1998) algorithms were used to transform Actigraph data in energy expenditure and metabolic equivalents (METs). The Troiano Adult (2008) algorithm was used to classify the activities into bouts and sedentary time. ActiLife exported Actigraph activity data into a spreadsheet with output including overall physical activity (minutes/day), non-wear time (hours), and time spent (minutes/day) engaged in sedentary behavior and light, moderate, and vigorous intensity physical activity.

**Supplementary B. Distribution of data for the primary outcome**

**Supplementary Figure 1. Histogram showing the distribution of overall healthcare utilization cost.**


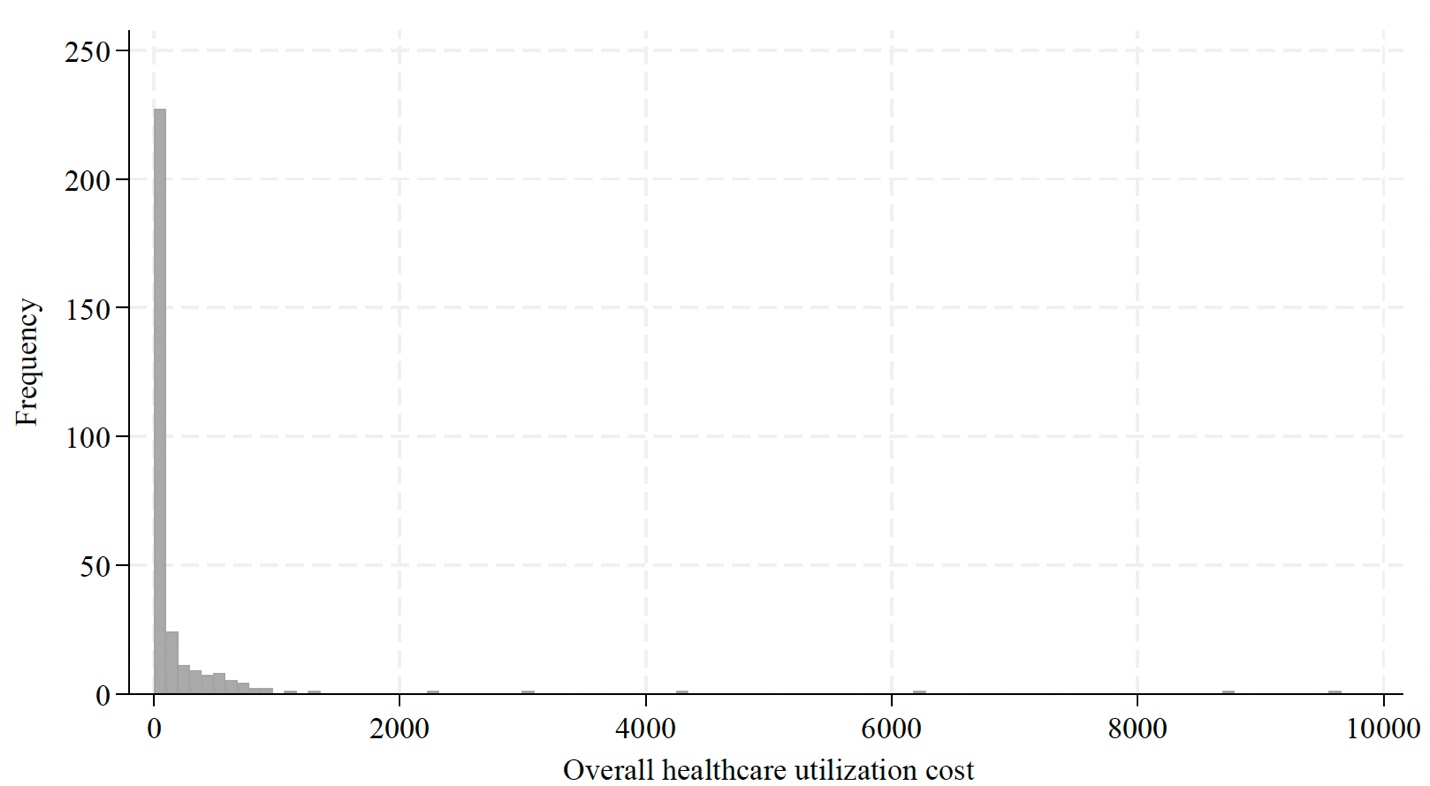


**Supplementary Figure 2. Lowess-smoothed residual plot assessing linearity of positive lifestyle behavior scale in the two-part cost model.**


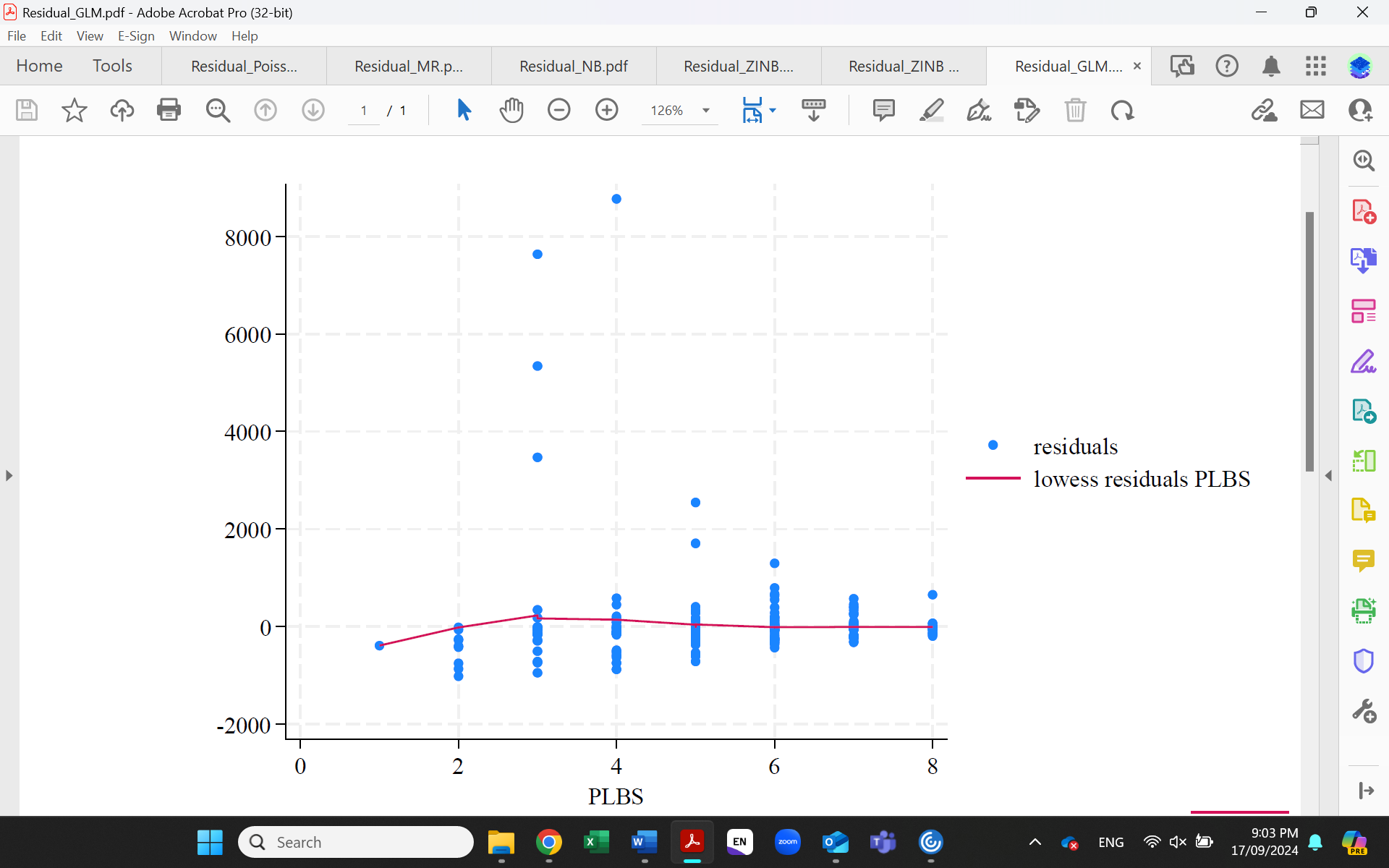


**Supplementary Table 1. Descriptions of the overall healthcare utilization cost (n: 307 participants).**

| **Costs** | **Median (IQR)** | **Mean (SD)^a^** |
| --- | --- | --- |
| Healthcare visit cost |  |  |
| General practitioner | 0 (0 – 0) | 15.93 (87.85) |
| Physiotherapist | 0 (0 – 0) | 67.48 (462.03) |
| Chiropractor | 0 (0 – 0) | 23.57 (131.06) |
| Emergency department | 0 (0 – 0) | 12.90 (186.22) |
| Surgical procedure | 0 (0 – 0) | 20.60 (207.67) |
| Other healthcare practitioners | 0 (0 – 0) | 41.33 (155.10) |
| Medication cost |  |  |
| Non-opioid medication | 0 (0 – 15.78) | 11.10 (22.17) |
| Weak opioid medication | 0 (0 – 0) | 18.19 (117.75) |
| Strong opioid medication | 0 (0 – 0) | 0.74 (7.54) |
| Antidepressant medication | 0 (0 – 0) | 0.52 (5.29) |

IQR: interquartile range, SD: standard deviation, n: total number of participants.

^a^ Median (IQR) was omitted in variables presenting frequently zero values. Mean (SD) was reported.

**Supplementary Table 2. Characteristic of the participants having overall healthcare utilization cost larger than AU$4,000 (n: 4 participants).**

| **Characteristic** | **Median (IQR)** |
| --- | --- |
| Age | 62.0 (41.4 – 76.3) |
| Sex (female) | 100% (n = 4) |
| Body Mass Index (kg/m^2^) | 25.7 (24.2 – 31.4) |
| Recent episode of LBP (yes)^a^ | 100% (n = 4) |
| LBP duration^b^ |  |
| 3 months or more | 100% (n = 4) |
| Depression (0 – 42)^c^ | 4 (1 – 7) |
| Anxiety (0 – 42)^c^ | 3 (0 – 7) |
| Stress (0 – 42)^c^ | 15 (8 – 16) |
| Positive lifestyle behavior scale | 3 (3 – 3.5) |
| Healthcare visit cost |  |
| General practitioner | 538.85 (165.80 – 829.00) |
| Physiotherapist | 1702.80 (1064.25 – 4576.28) |
| Chiropractor | 0 (0 – 638.55) |
| Emergency department | 0 (0 – 1583.90) |
| Surgical procedure | 2107.67 (2107.67 – 2107.67) |
| Other healthcare practitioners | 159.87 (55.18 – 272.72) |
| Medication cost |  |
| Non-opioid medication | 94.68 (47.34 – 181.47) |
| Weak opioid medication | 742.69 (512.20 – 1216.48) |
| Strong opioid medication | 42.38 (0 – 84.75) |
| Antidepressant medication | 13.43 (0 – 53.70) |

IQR: interquartile range, n: total number of participants.

^a^ Recent episode of LBP is defined as experiencing low back pain ≤4 weeks prior to completion of baseline assessment.

^b^ Data on LBP duration were collected when individuals reported recent episode of LBP.

^c^ 21-item Depression Anxiety Stress Scale; each of the three domains range from 0–42, with higher scores representing higher levels of each domain.

**Supplementary C. Results of the two-part model and margins prediction**

**Supplementary Table 3. The association between the positive lifestyle behavior scale, covariates and overall costs, medication costs and healthcare visit costs due to low back pain.**

| **Variables** | **Logit (n = 307)** |  | **GLM (n = 173)** |  |  |
| --- | --- | --- | --- | --- | --- |
|  | **OR (95% CI)** | ***p*** | **CR (95% CI)** | ***p*** |  |
|  | **Overall healthcare costs^a^** | | | |  |
| Positive lifestyle behavior scale^b^ | 0.783 (0.654 to 0.938) | 0.008 | 0.771 (0.641 to 0.927) | 0.006 |  |
| Sex (male) ^c^ | 0.451 (0.246 to 0.827) | 0.010 | 0.243 (0.110 to 0.539) | <0.001 |  |
| Age | 1.016 (0.995 to 1.038) | 0.15 | 1.004 (0.983 to 1.026) | 0.71 |  |
| Recent episode of LBP^d^ | 3.231 (1.947 to 5.363) | <0.001 | 3.081 (1.872 to 5.072) | <0.001 |  |
| Depression | 0.988 (0.929 to 1.051) | 0.71 | 0.991 (0.961 to 1.022) | 0.57 |  |
| Anxiety | 1.043 (0.930 to 1.169) | 0.47 | 0.955 (0.909 to 1.003) | 0.065 |  |
| Stress | 1.017 (0.965 to 1.071) | 0.53 | 1.042 (0.991 to 1.094) | 0.11 |  |
|  | **Medication costs^a^** | | | |  |
| Positive lifestyle behavior scale^b^ | | 0.789 (0.662 to 0.940) | 0.008 | 0.754 (0.650 to 0.874) | <0.001 |
| Sex (male) ^c^ | | 0.555 (0.304 to 1.012) | 0.055 | 0.504 (0.372 to 0.683) | <0.001 |
| Age | | 1.024 (1.003 to 1.045) | 0.022 | 1.028 (1.010 to 1.046) | 0.002 |
| Recent episode of LBP^d^ | | 2.609 (1.578 to 4.314) | <0.001 | 2.136 (1.559 to 2.924) | <0.001 |
| Depression | | 1.004 (0.948 to 1.063) | 0.89 | 1.004 (0.963 to 1.048) | 0.85 |
| Anxiety | | 1.017 (0.930 to 1.112) | 0.71 | 0.949 (0.898 to 1.003) | 0.066 |
| Stress | | 1.037 (0.988 to 1.088) | 0.15 | 1.077 (1.034 to 1.122) | <0.001 |
|  | **Healthcare visit costs^a^** | | | |  |
| Positive lifestyle behavior scale^b^ | | 0.943 (0.788 to 1.129) | 0.52 | 0.727 (0.614 to 0.860) | <0.001 |
| Sex (male) ^c^ | | 0.209 (0.097 to 0.451) | <0.001 | 0.458 (0.163 to 1.286) | 0.14 |
| Age | | 0.996 (0.976 to 1.017) | 0.73 | 1.004 (0.983 to 1.026) | 0.70 |
| Recent episode of LBP^d^ | | 3.321 (1.798 to 6.135) | <0.001 | 2.145 (1.393 to 3.302) | 0.001 |
| Depression | | 0.988 (0.933 to 1.046) | 0.68 | 0.995 (0.971 to 1.021) | 0.72 |
| Anxiety | | 1.045 (0.975 to 1.121) | 0.21 | 0.934 (0.892 to 0.978) | 0.004 |
| Stress | | 1.000 (0.952 to 1.050) | 1.0 | 1.048 (0.995 to 1.104) | 0.080 |

OR: odds ratio, CR: cost ratio, CI: confidence interval, p: probability, LBP: low back pain.

^a^ All analyses were adjusted for sex, age, depression, anxiety, stress, and recent episode of LBP at baseline.

^b^ Positive lifestyle behavior score was treated as a continuous variable in these analyses.

^c^ Female sex was used as the reference in the analyses.

^d^ Recent episode of LBP is defined as experiencing low back pain ≤4 weeks prior to completion of baseline assessment.

**Supplementary Table 4. Margins predictions for overall costs, medication costs and healthcare visit costs.**

|  | **Margins predictions of overall healthcare costs** | | | |
| --- | --- | --- | --- | --- |
| **Positive lifestyle behavior scale** | **Cost** | **SE** | ***p*** | **95% CI** |
| 0 | 946.49 | 551.11 | 0.086 | -133.66 to 2026.65 |
| 1 | 707.41 | 353.32 | 0.045 | 14.92 to 1399.91 |
| 2 | 525.38 | 220.35 | 0.017 | 93.51 to 957.26 |
| 3 | 387.38 | 133.29 | 0.004 | 126.14 to 648.61 |
| 4 | 283.30 | 78.70 | <0.001 | 129.05 to 437.54 |
| 5 | 205.30 | 47.20 | <0.001 | 112.79 to 297.81 |
| 6 | 147.30 | 31.79 | <0.001 | 85.00 to 209.60 |
| 7 | 104.54 | 25.62 | <0.001 | 54.32 to 154.76 |
| 8 | 73.34 | 22.72 | 0.001 | 28.81 to 117.87 |
|  | **Margins predictions of medication costs** | | | |
| **Positive lifestyle behavior score** | **Cost** | **SE** | ***p*** | **95% CI** |
| 0 | 149.42 | 123.09 | 0.23 | -91.83 to 390.67 |
| 1 | 107.96 | 77.38 | 0.16 | -43.71 to 259.62 |
| 2 | 77.42 | 47.53 | 0.10 | -15.73 to 170.56 |
| 3 | 55.05 | 28.51 | 0.054 | -0.83 to 110.94 |
| 4 | 38.80 | 16.84 | 0.021 | 5.78 to 71.81 |
| 5 | 27.08 | 10.08 | 0.007 | 7.31 to 46.84 |
| 6 | 18.70 | 6.49 | 0.004 | 5.98 to 31.42 |
| 7 | 12.78 | 4.71 | 0.007 | 3.54 to 22.02 |
| 8 | 8.64 | 3.75 | 0.021 | 1.29 to 15.99 |
|  | **Margins predictions of healthcare visit costs** | | | |
| **Positive lifestyle behavior score** | **Cost** | **SE** | ***p*** | **95% CI** |
| 0 | 1028.44 | 652.33 | 0.12 | -250.09 to 2306.98 |
| 1 | 725.51 | 396.94 | 0.068 | -52.49 to 1503.51 |
| 2 | 511.44 | 236.35 | 0.030 | 48.20 to 974.69 |
| 3 | 360.27 | 137.49 | 0.009 | 90.79 to 629.76 |
| 4 | 253.61 | 78.69 | 0.001 | 99.37 to 407.84 |
| 5 | 178.39 | 45.91 | <0.001 | 88.42 to 268.36 |
| 6 | 125.39 | 29.73 | <0.001 | 67.12 to 183.67 |
| 7 | 88.08 | 22.81 | <0.001 | 43.36 to 132.79 |
| 8 | 61.82 | 19.52 | 0.002 | 23.57 to 100.07 |

SE: Standard Error, *p*: p value, CI: Confidence Interval.
